# Improved survival in patients enrolled promptly into remote monitoring following cardiac implantable electronic device implantation

**DOI:** 10.1007/s10840-016-0112-y

**Published:** 2016-02-10

**Authors:** Suneet Mittal, Jonathan P. Piccini, Jeff Snell, Julie B. Prillinger, Nirav Dalal, Niraj Varma

**Affiliations:** Valley Health System of New York and New Jersey, 223 North Van Dien Avenue, Ridgewood, NJ 07450 USA; Duke University Medical Center, Durham, NC USA; Data Informs, LLC, Sylmar, CA USA; St. Jude Medical, Inc., Sylmar, CA USA; Cleveland Clinic, Cleveland, OH USA

**Keywords:** Cardiac implantable electronic device, Remote monitoring, Survival, Time to enrollment

## Abstract

**Purpose:**

Guidelines advocate remote monitoring (RM) in patients with a cardiac implantable electronic device (CIED). However, it is not known when RM should be initiated. We hypothesized that prompt initiation of RM (within 91 days of implant) is associated with improved survival compared to delayed initiation.

**Methods:**

This retrospective, national, observational cohort study evaluated patients receiving new implants of market-released St. Jude Medical™ pacemakers (PM), implantable cardioverter defibrillators (ICD), and cardiac resynchronization therapy (CRT) devices. Patients were assigned to one of two groups: an “RM Prompt” group, in which RM was initiated within 91 days of implant; and an “RM Delayed” group, in which RM was initiated >91 days but ≤365 days of implant. The primary endpoint was all-cause mortality.

**Results:**

The cohort included 106,027 patients followed for a mean of 2.6 ± 0.9 years. Overall, 47,014 (44 %) patients had a PM, 31,889 (30 %) patients had an ICD, 24,005 (23 %) patients had a CRT-D, and 3119 (3 %) patients had a CRT-P. Remote monitoring was initiated promptly (median 4 weeks [IQR 2, 8 weeks]) in 66,070 (62 %) patients; in the other 39,957 (38 %) patients, RM initiation was delayed (median 24 weeks [IQR 18, 34 weeks]). In comparison to delayed initiation, prompt initiation of RM was associated with a lower mortality rate (4023 vs. 4679 per 100,000 patient-years, *p* < 0.001) and greater adjusted survival (HR 1.18 [95 % CI 1.13–1.22], *p* < 0.001).

**Conclusions:**

Our data, for the first time, show improved survival in patients enrolled promptly into RM following CIED implantation. This advantage was observed across all CIED device types.

## Introduction

Hundreds of thousands of cardiac implantable electronic devices (CIEDs) are implanted worldwide annually [1, 2]. As indications have expanded and technology has improved, the incidence of device implantation has increased. Guidelines have been developed to guide the frequency and method of follow-up for CIED patients [1, 3]. It is accepted that all patients should be seen in-person within 2–12 weeks of device implantation. Subsequently, patients have typically been seen quarterly for in-person evaluation.

The technology now exists for physicians to remotely access data from their patients’ CIEDs, including diagnostic data (arrhythmia burden, transthoracic impedance) and lead parameters (pacing impedance, capture threshold). In fact, it has been shown that CIED patients who are monitored remotely have improved survival [4–6]. While the majority of prior work has focused on patients implanted with an ICD or CRT-D, the survival benefit is maintained across all CIED types (including pacemakers) and is enhanced by high adherence to RM [7]. Thus, current guidelines advocate remote monitoring (RM) over calendar-based in-person only device follow-up [3].

However, an unresolved issue is the timing of RM enrollment and activation relative to device implantation itself. Several device manufacturers now offer “Point of Care” (POC) pairing, in which a patient’s newly implanted CIED is synchronized with their remote transmitter prior to hospital discharge or at the first in-office visit. In a small pilot study of radiofrequency-enabled pacemaker patients, POC pairing was associated with increased RM compliance at 2 months post-implant [8]. Whether prompt initiation of RM translates into improved clinical outcomes is unknown. We hypothesized that prompt initiation of RM, defined as within 91 days of CIED implantation, is associated with improved survival compared to delayed initiation. The aim of this study was to assess this hypothesis in a large national cohort of CIED patients.

## Methods

### Study design and patient selection

**Fig. 1:**
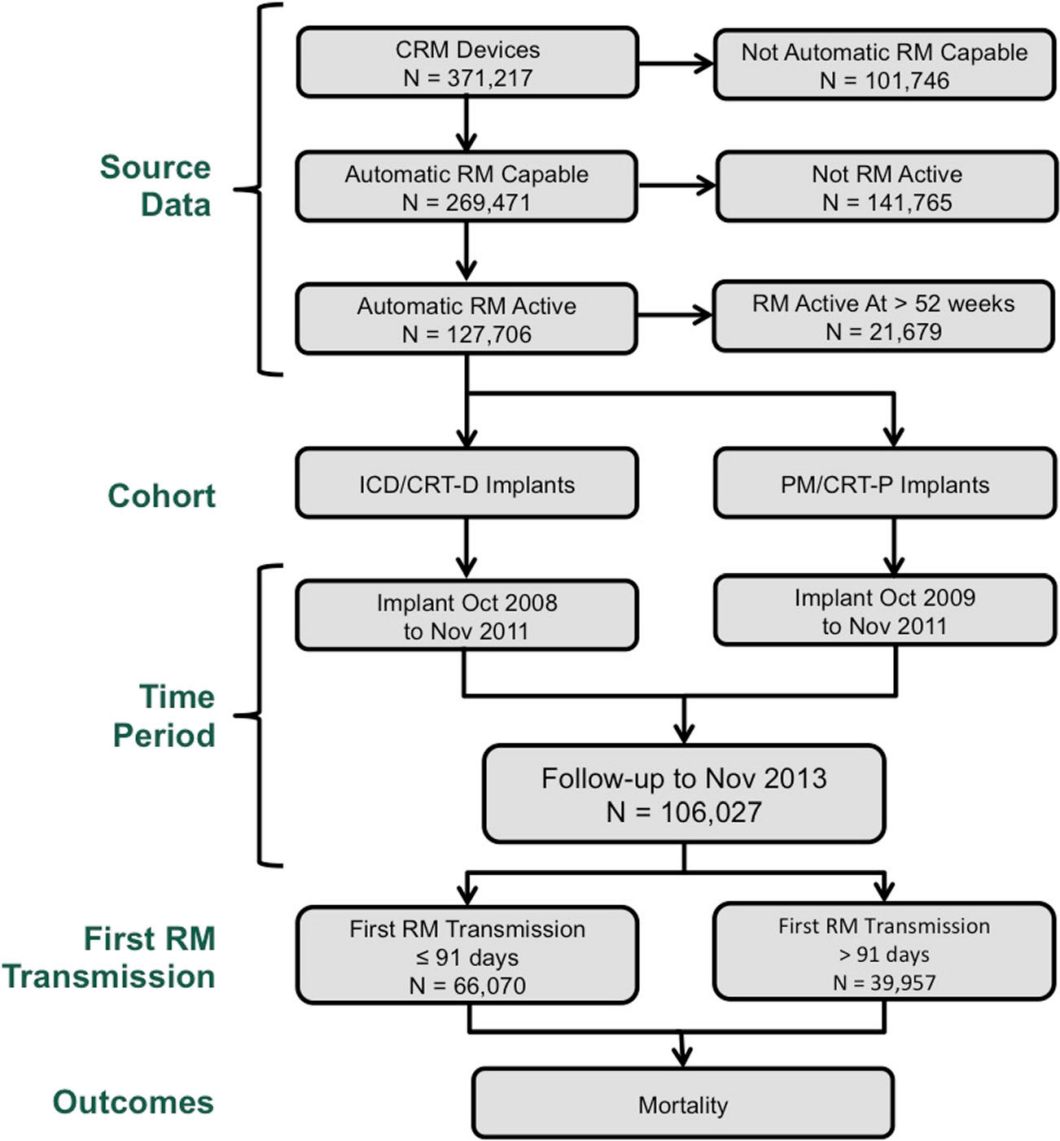
Flowchart of patients

This retrospective, national, observational cohort study evaluated patients receiving new implants of a market-released St. Jude Medical™ radiofrequency-enabled pacemaker (PM), implantable cardioverter defibrillator (ICD), cardiac resynchronization therapy (CRT)-pacemaker (CRT-P), or CRT-defibrillator (CRT-D). To assess the impact of RM initiation on outcome, patients whose implanted device did not support automatic (“wandless”) daily monitoring were excluded (not automatic RM capable, Fig. 1). Among “automatic RM capable” patients who were active on RM, an index event (RM_0_) was defined as the time of first connection between a patient’s Merlin@home monitor and the central server. Only “automatic RM active” patients in whom RM_0_ occurred within 1 year of implant were included in the study. The remaining patients with an ICD or CRT-D device implanted between October 2008 and November 2011 and a PM or CRT-P device implanted between October 2009 and November 2011 comprised the study cohort. Among these, patients enrolled in another clinical trial, or with follow-up time <90 days, were excluded. Included patients were followed until death, device replacement, or device removal through November 2013.

### Data acquisition

Study data were obtained from four sources: device implant registration from St. Jude Medical, Inc. (Sylmar, CA), Merlin.net™ remote monitoring system, the 2012 American Community Survey (ACS) of the US Census, and the US Social Security Death Index (SSDI) Master File. Age at implant, gender, patient ZIP code, device model number, date of implant, and follow-up duration were ascertained using manufacturer device tracking data. De-identified data from weekly Merlin.net™ maintenance transmissions were linked to implant registration data to determine RM status. The date of death was determined from the SSDI, a database of internal records from the US Social Security Administration Death Master File, with all death records through November 30, 2013. The SSDI contains records of >94 million deceased individuals in the US and maintains high accuracy in determination of mortality status, with increased sensitivity in patients >65 years [9–11]. We added death reports made directly to the device manufacturer’s US tracking system by healthcare providers or family members (accounting for <1 % of deaths) through November 30, 2013. Socioeconomic data was gathered from the 2012 ACS by individual ZIP code tabulation area (ZCTA). The ACS ZCTA-based data was then linked to individual patient ZIP codes for the following data in percent of population in ZCTA, except as noted: 4-year college degree, median income, below poverty level, Supplemental Nutrition Assistance Program (SNAP) recipient, telephone or cell phone service, employment status, labor force participation rate (LFPR), civilian population proportion, healthcare insurance, racial population proportions (White, Black, American Indian, Asian, two races, other), and total urban/rural classification of population counts. The urban percentage for a region was computed as the ratio of urban to total population counts.

### Determination of time to remote monitoring

Among automatic RM active patients, the time to RM was defined as the number of days between implant and RM_0_. To determine whether the timing of RM initiation affects outcome, automatic RM active patients were assigned to one of two groups based on the computed time to RM. Patients with time to RM ≤ 91 days comprised the “RM Prompt” group, and those with 91 days < time to RM ≤ 365 days constituted the “RM Delayed” group. A pre-determined cutoff of 91 days was selected based on the 2008 HRS/EHRA expert consensus guideline that all CIED patients be seen in person within 12 weeks of device implant [1].

### Statistical analysis

The primary endpoint for this study was all-cause mortality. Mortality was determined using unadjusted mortality incidence rates and adjusted survival via Cox proportional hazards survival models. The mortality incidence rate ratio (RM Delayed/RM Prompt) and 95 % confidence intervals (CI) were determined from the patient deaths and the computed follow-up duration within each group. All-cause survival was compared among patients within the RM Prompt and RM Delayed groups using multivariable Cox proportional hazards modeling with stratification on age and covariates of gender, device type (for groupings with >1 device type), and the RM predictor census variables. Covariates were evaluated between the RM Prompt and RM Delayed groups using logistic regression and stepwise backward elimination for *p* values ≤ 0.2. These variables were then used for adjustment in the survival regression to determine the Cox proportional hazard ratio (HR) and 95 % CI. Follow-up duration was calculated for each patient as the time from device implant until device explant, device replacement, death, or end of study surveillance.

All statistical analyses were performed with Revolution R Open 3.2.1. Patient demographics were assessed as mean and standard deviation, median and quartiles, or count and proportion. The *p* value for means comparison was Student’s *t* test and for counts was Chi square.

## Results

The study cohort included 106,027 patients. The mean age of the patients was 71 ± 13 years, and 68,159 (64 %) patients were male. Overall, 47,014 (44 %) patients had a PM, 31,889 (30 %) patients had an ICD, 24,005 (23 %) patients had a CRT-D, and 3119 (3 %) patients had a CRT-P device. Patients were followed for a mean of 2.6 ± 0.9 years. By design, all patients were enrolled in automatic (wandless) RM. The median time to RM for the entire cohort was 8 weeks (IQR 4, 20 weeks).

**Fig. 2:**
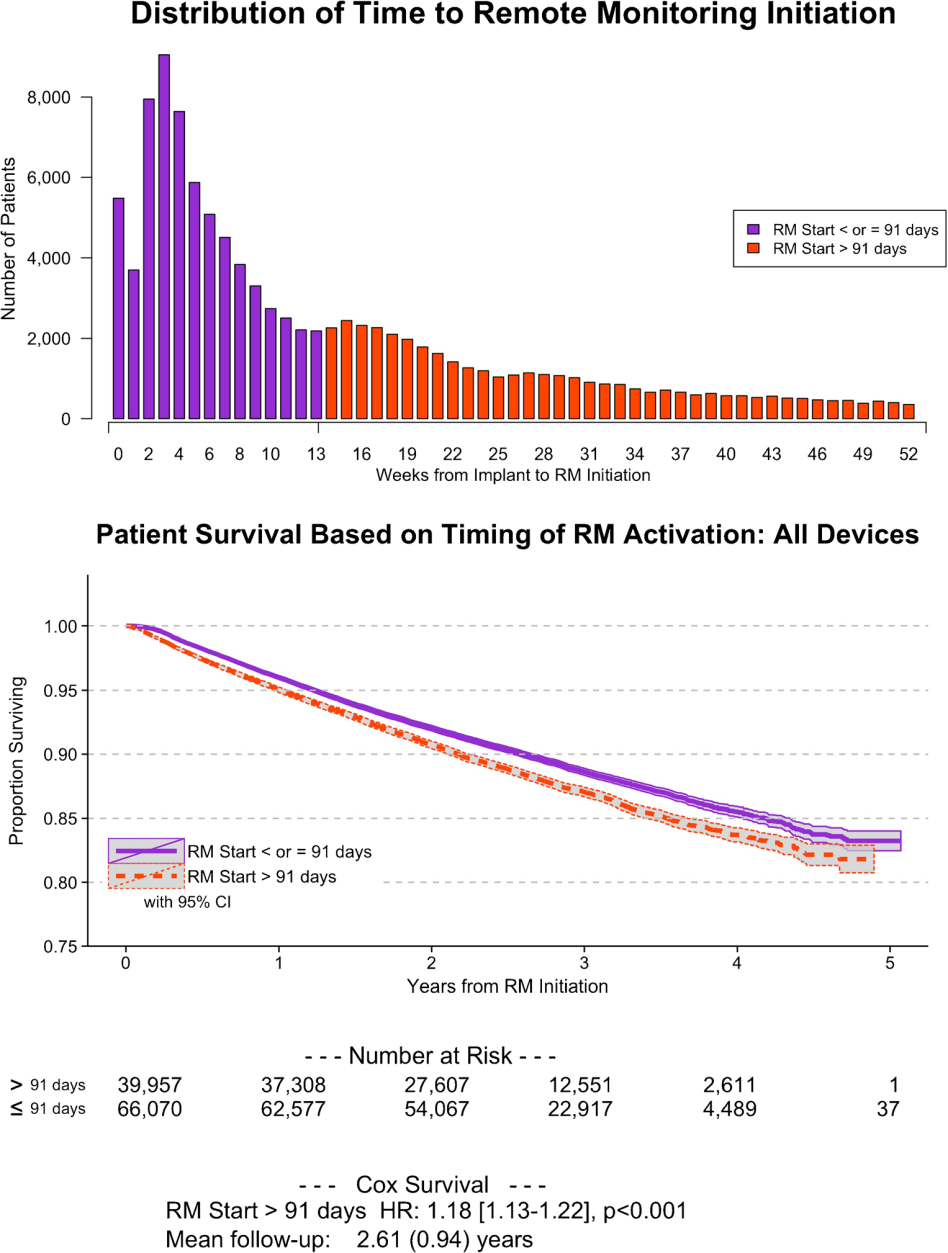
Timing of remote monitoring initiation and its impact on patient survival. In this study, remote monitoring was promptly initiated (≤91 days post-implant) in 66,070 (62 %) patients; in the other 39,957 (38 %) patients, RM initiation was delayed (>91 days) within the first year post-implant (*top*). Early activation of remote monitoring was associated with improved patient survival for all devices (*bottom*)

Remote monitoring was initiated within 91 days of implant in 66,070 (62 %) patients (Table 1). In these patients, RM was initiated at a median of 4 weeks (IQR 2, 8 weeks) following device implantation. There were 39,957 (38 %) patients in whom RM was initiated after 91 days of device implantation (but still within the first year). In these patients, RM was initiated at a median of 24 weeks (IQR 18, 34 weeks). For the overall cohort, the number of weeks between implant and RM_0_ is illustrated in the Fig. 2. This distribution was similar across all CIED device types; however, there were numerically a higher proportion of patients with delayed RM initiation in the ICD and CRT-D cohorts (43 and 42 %, respectively) compared to the PM and CRT-P cohorts (32 and 35 %, respectively). Patients (matched to individual ZIP codes) in both groups were similar with respect to all socio-demographic characteristics assessed in this study (Table 2).

**Table 1:** Comparison of patients based on time to enrollment into remote monitoring following CIED device implantation

	RM_0_ ≤ 91 days, *n* = 66,070	RM_0_ > 91 days, *n* = 39,957
Age, years	72 ± 13	71 ± 13
Sex, male	42,207 (64 %)	25,952 (65 %)
Device type		
CRT-pacemaker	2030 (3 %)	1089 (3 %)
CRT-defibrillator	13,971 (21 %)	10,034 (25 %)
ICD	18,300 (28 %)	13,589 (34 %)
Pacemaker	31,769 (48 %)	15,245 (38 %)
Follow-up, years	2.7 ± 0.9	2.5 ± 1.0
Time to first RM, weeks [IQR]	4 (2, 8)	24 (18, 34)

Due to the large sample size, comparison between groups yields differences that are very small in magnitude and not clinically meaningful but statistically significant. Thus, the *p* values are not shown

*CRT* cardiac resynchronization therapy, *ICD* implantable cardioverter-defibrillator, *IQR* interquartile range, *RM* remote monitoring, *RM*_*0*_ number of days between CIED implant and RM initiation

**Table 2:** Socio-demographic data

ZIP code-linked data^a^	RM_0_ ≤ 91 days, *n* = 66,070	RM_0_ > 91 days *n* = 39,957
Bachelor’s degree	26 (15)	26 (15)
Median income	54 (21)	55 (22)
Below poverty line	14 (8)	13 (8)
Have telephone	98 (2)	98 (2)
Receive SNAP	1 (1)	1 (1)
Uninsured	14 (7)	14 (7)
Residence: urban	71 (36)	73 (35)
Not in labor force (1-LPFR)	37 (9)	37 (9)
Unemployed	9 (4)	9 (4)
Civilian	62 (9)	62 (8)
Race: White	80 (19)	79 (20)
Race: Black	11 (17)	12 (18)
Race: Indian (American)	1 (3)	1 (3)
Race: Asian	3 (6)	3 (06)
Race: two races	2 (2)	2 (2)
Race: other	3 (5)	3 (5)

^a^Values reported as mean (standard deviation). All parameters in this section were measured as percent in ZIP code except median income, which was thousands of dollars in ZIP code. There are 16,385 unique ZIPs in the “RM Early” group and 13,683 in the “RM Delayed” group. Due to the large sample size, comparison between groups yields differences that are very small in magnitude and not clinically meaningful but statistically significant. Thus, the *p* values are not shown

**Fig. 3:**
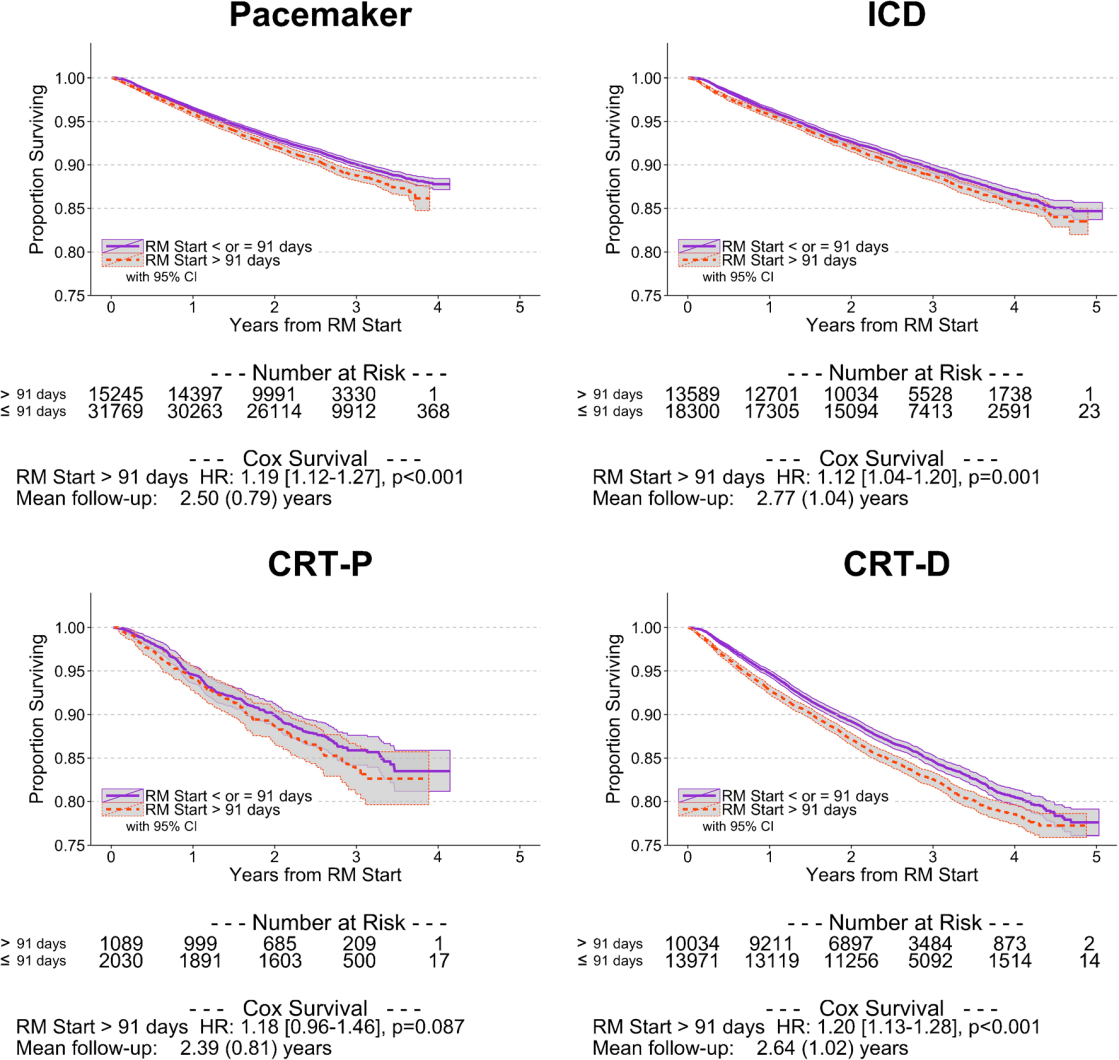
Survival in patients who did or did not promptly enroll in remote monitoring after device implantation, stratified by type of cardiac implantable electronic device

In comparison to patients with delayed initiation of RM, patients in whom RM was initiated promptly had a lower mortality incidence rate (4023 vs. 4679 per 100,000 patient-years, *p* < 0.001) (Table 3). This relationship held across all CIED types. Unadjusted mortality incidence rates were highest in the CRT population. The adjusted survival was significantly greater in patients in whom RM was started promptly (HR 1.18 [95 % CI 1.13–1.22], *p* < 0.001, Fig. 2). The magnitude of this relationship was similar across each device type, but was greatest for patients implanted with CRT-D devices (HR 1.20 [95 % CI 1.13–1.28], *p* < 0.001, Fig. 3).

**Table 3:** Mortality rate table

	Mortality incidence rate per 100,000 patient-years		Mortality incidence rate ratio^a^	*p* value
	RM_0_ ≤ 91 days	RM_0_ > 91 days		
All devices	4,023	4,679	1.2	<0.001
CRT-defibrillator	5,539	6,473	1.2	<0.001
CRT-pacemaker	5,135	5,898	1.2	<0.001
ICD	3,668	4,002	1.1	<0.001
Pacemaker	3,480	4,010	1.2	<0.001

^a^MIRR = MIR_Delayed_/MIR_Prompt_

## Discussion

Prior prospective and observational mega-cohort studies have demonstrated an association between remote monitoring of CIED patients and improved survival [4–7]. Our data, for the first time, show the importance of prompt enrollment into remote monitoring following CIED implantation. Patients with RM activation within 3 months of CIED implantation had an 18 % increased survival during a mean follow-up of 2.6 years. This advantage was observed across all CIED device types, highlighting the consistency and relevance of these findings for all patients with cardiac rhythm devices.

In the current study, CRT-D patients exhibited the largest survival benefit with prompt initiation of RM. While the precise mechanism for this effect is not yet fully understood, our results align with findings from the IN-TIME trial. This randomized trial showed ICD and CRT-D patients with heart failure in whom RM was utilized in the year post-device implant had a significantly lower likelihood of the composite endpoint of all-cause death, overnight hospital admission for heart failure, change in NYHA class, and change in patient global self-assessment [6]. Three mechanisms were proposed for the observed benefit: (1) early detection of ventricular and atrial tachyarrhythmias, (2) early recognition of suboptimal device function such as low percent biventricular pacing and inappropriate shocks, and (3) patient interview prompted by remote monitoring, which occasionally revealed symptomatic worsening or noncompliance to drugs. Since patients most vulnerable to repeated heart failure hospitalizations have the highest mortality, prompt correction of potentially destabilizing conditions (enabled by RM) is likely to improve future survival [12].

Importantly, the association between prompt initiation of RM and improved survival was observed across all CIED types. In ICD patients, RM has been associated with a lower likelihood of inappropriate and appropriate shocks [13], reduced time from event to clinical decision [14], and earlier detection of arrhythmias and hardware malfunction, initiating both surgical and noninvasive (reprogramming, introduction of antiarrhythmics) interventions [15, 16]. This effect was maintained even when the analysis was limited to the first 3 months post-implant, suggesting that early RM promotes the detection of actionable events immediately following ICD implant [17]. In PM patients, early detection of atrial arrhythmias via RM may result in alteration of management that translates to fewer strokes and associated hospitalizations [18].

Despite these data and guideline recommendations to incorporate RM in the care of all CIED patients, many challenges remain, and between a third and half of all patients with an implanted CIED never activate RM [19]. Identifying the barriers to early RM initiation is paramount to developing clinical solutions and improving outcomes for CIED patients. The PREDICT-RM study found that RM enrollment is dictated primarily by the local practice at the institution at which the CIED is implanted [20]. Additional patient-related variables that influenced RM activation included race, ethnicity, health insurance status, geographic location, age, procedure-related indications and adverse events, and the presence of co-morbidities such as lung disease, renal dysfunction, hyponatremia, and atrial arrhythmias [20]. PREDICT-RM did not, however, investigate the timing of RM initiation, and it is unknown whether similar factors mediate prompt vs. delayed RM activation.

It has been postulated that initiation of RM may be more likely in patients who are generally more compliant with recommended therapy, such as diet, medications, and/or contact with their physicians. However, it is also possible that RM actually induces a “healthy user effect.” In particular, CIED patients who are remotely monitored are less likely to be lost to follow-up and more likely to adhere to in-person follow-up [21]. This effect may be amplified over time in patients who commit promptly to RM, although the absence of patient and physician characteristics in the current dataset does not allow for investigation into this hypothesis.

Data from prior randomized trials, observational studies, and meta-analyses indicate that routine use of RM in CIED patients is associated with a reduction in healthcare utilization costs [14, 22–25]; however, none of these studies have investigated the impact of early RM initiation on healthcare costs. Given the improved clinical outcomes associated with prompt post-implant RM activation, one may speculate there could be an associated economic benefit, but further investigation is required to draw this connection.

Our data included only patients who initiated wireless RM within 1 year of CIED implant, with the intent of mitigating potential biases associated with unusual follow-up patterns. The increased survival associated with prompt initiation of RM, irrespective of CIED type, suggests that we may have an opportunity to improve outcomes by simply changing our post-CIED implant workflow. Our data support current consensus guidelines, which suggest enrolling patients into RM within 2 weeks of device implantation [3]. The guidelines further note that by activating RM prior to hospital discharge, also known as “Point of Care” pairing, a clinic may confirm successful transmission or troubleshoot technical/compliance issues at the first in-office device check [3]. This strategy has been previously associated with increased RM compliance in pacemaker patients [8]. We show, for the first time, that prompt initiation of RM is associated with improved survival across all CIED device types.

### Limitations

There are several limitations to this retrospective, observational study. First, it is not possible to determine why some patients promptly engaged with RM post-CIED implant whereas others delayed initiation. Additionally, it is not possible to know whether delay was related to failure to enroll patients promptly into RM or failure of an enrolled patient to activate their remote monitor. We also do not have information regarding the timing or frequency of in-clinic device follow-up during this first year. Finally, it is not possible to ascertain the mechanism of the survival benefit observed in the patients enrolled early into RM. We do not have information about device or arrhythmia-related issues that may have been detected early post-device implant in the patients who were promptly enrolled into RM. Recognition and intervention to correct these issues may have contributed to the observed survival benefit in our cohort. Similarly, while we did adjust for patient age and gender, minimal clinical characteristics are available for our cohort, and the potential for patient or physician bias cannot be excluded.

### Conclusions

Following CIED implantation, all patients should be offered enrollment into RM. Patients should be educated on the association between RM and improved outcomes (including survival) and be reminded that the benefit appears to begin early post-CIED implantation. Subsequent efforts can then be directed to ensure a high adherence to RM over time, which is necessary to derive the maximal benefit with this technology.
